# Diffuse telangiectasia of the colon: A case report

**DOI:** 10.1097/MD.0000000000021106

**Published:** 2020-08-21

**Authors:** Jun-An Li, Li-Li Zhong, Bo Li, Dong-Qiang Jiang, Yin-Long Zhao

**Affiliations:** a Gastroenterology and Center of Digestive Endoscopy; b Jilin Provincial Key Laboratory on Molecular and Chemical Genetic; c Cardiovascular Surgery; d Department of Nuclear Medicine, The Second Hospital of Jilin University, Changchun, Jilin, P. R. China.

**Keywords:** Bleeding, Colonic telangiectasia, Colonoscopy, Gastrointestinal tract

## Abstract

**Rationale:**

Colonic telangiectasia, also known as colonic angiodysplasia, refers to arteriovenous malformations that occur in the colon, which are common vascular lesions in the GI tract.

**Patient concerns:**

We report a patient, who was admitted to our hospital for colonoscopy.

**Diagnoses:**

Under a microscope, all the segments of the whole colon and the varicose veins showed multiple flaky spider-like telangiectasia changes. The blood vessels were radially distributed and converged in the center. The largest blood vessel was about 10 mm in diameter and had a smooth surface with no ulcers, erosion, or bleeding.

**Interventions:**

It was recommended that the patient undergo a capsule endoscopy to examine small intestine.

**Outcomes:**

The patient did not agree to endoscopy for personal reasons. During the follow-up half a year later, the patient had no melena with normal range of hemoglobin and red blood cell counts. The fecal occult blood test came out negative.

**Lessons:**

While the etiology of colonic telangiectasia remains unclear, it is common in the elderly, and is more associated with geriatric conditions and diseases, especially atherosclerotic diseases. Patients who are diagnosed with colonic telangiectasia but are asymptomatic, do not need further treatment. It is usually recommended to monitor the color of stool and check the hemoglobin and fecal occult blood regularly. Colonoscopy is the main method of diagnosis of colonic telangiectasia, and the positive rate is greater than 90%. This procedure should be performed when there is no bleeding or a small amount of bleeding.

## 1 INTRODUCTION

Colonic telangiectasia, also known as colonic angiodysplasia, refers to arteriovenous malformations that occur in the colon, which are the common vascular lesions in the gastrointestinal (GI) tract.^[1]^ The blood vessels become enlarged and fragile in the colon, causing lower GI hemorrhage in the elderly.^[1–4]^ However, the vast majority of the patients do not exhibit any clinical symptoms and, therefore, misdiagnosis and mistreatment is common.^[5]^ In addition, only a small number of patients with repeated episodes of painless lower gastrointestinal bleeding are discovered incidentally during colonoscopy.^[6,7]^ The cause of colonic telangiectasia is not known but it is thought that ageing and degeneration of blood vessels play a critical role.^[2,8]^ While most reported cases have shown that colonic telangiectasia predominantly affects the cecum and right side of the colon,^[6,9]^ it may also affect any part of the large bowel.^[9]^ Furthermore, it has been reported that the incidence of colonic telangiectasia is about 0.8% in healthy people who received colonoscopy screening in the US.^[9,10]^ However, the incidence of colonic telangiectasia in China is not clear due to the paucity of available data. This article reports a rare case of colonic telangiectasia, which was distributed throughout the colon.

## 2 CASE PRESENTATION

The patient, a 55 years old female, was admitted to a local hospital for “black stool for 2 months which aggravated 1 day back.” The patient was physically healthy and denied any history of liver disease. About 2 months ago, patient had black stool with no obvious causes. Patient showed no obvious abdominal pain, no abdominal distension, no nausea or vomiting, no fever, no diarrhea, no mucous stool with pus and blood, and no rectal tenesmus. Because it did not significantly affect the patient's daily life, she was not admitted to the hospital. One day ago, the black stool aggravated, and the color of the stool gradually turned dark red. At the same time, it was accompanied by dizziness, fatigue, and palpitation. She was then treated at a local hospital and admitted to the hospital with “gastrointestinal bleeding.”

**Figure 1:**
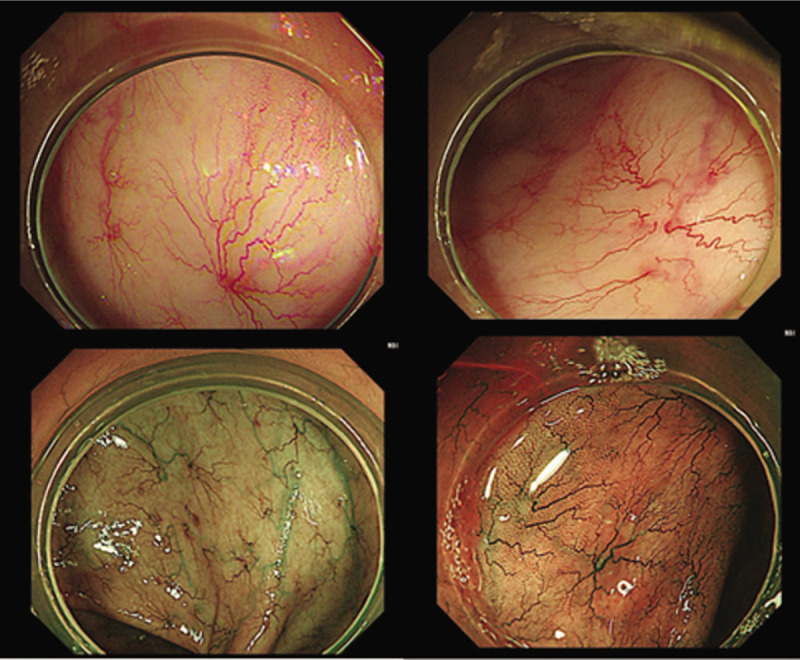
In colonoscopy, segments of the whole colon and the varicose veins showed multiple flaky spider-like telangiectasia changes. The blood vessels were radially distributed and converged in the center.

Admission examination showed: blood pressure of 80/60 mm Hg, heart rate of 118 beats/min, no abnormalities in the auscultation of the lungs, no murmur in heart, pale appearance to the palpebral conjunctiva, no purpura seen on face and tongue, spider angioma or telangiectasia, flat abdominal, no abdominal varicose veins or purpura, no gastrointestinal and peristaltic waves, no abdominal tenderness, no muscle tension and rebound tenderness, no palpable abdominal mass, and negative abdominal shifting dullness. The bowel sound was 6 times/min. RBC was 2.7 × 10^12^/L, Hb was 62 g/L, HCT was 21.5%, and ferritin was 51.644. All results were within the normal range of reference values. The tests for hepatitis virus series were all negative, and liver function and kidney function were normal. Gastroscopic examination showed chronic gastritis - non-atrophic and no obvious abnormalities in CT scans of the whole abdomen and pelvis. After transfusion and symptomatic treatment, the patient was discharged. After discharging from the hospital, stools were routinely monitored. All examination showed yellow soft stools, and the fecal occult blood test was negative. In order to find out the cause of bleeding, patient was admitted to our hospital, and colonoscopy was performed (Figure 1). Under microscope, all the segments of the whole colon and the varicose veins showed multiple flaky spider-like telangiectasia changes. The blood vessels were radially distributed and converged in the center. The largest blood vessel 1 was about 10 mm in diameter and had a smooth surface with no ulcers, erosion, or bleeding. It was recommended that the patient undergo a capsule endoscopy to examine the small intestine. The patient did not agree because of personal reasons. During the follow-up half a year later, the patient had no melena with normal range of hemoglobin and red blood cell counts. Fecal occult blood test also turned out to be negative.

## 3 DISCUSSION

The etiology of colonic telangiectasia is still unclear. It is common in the elderly, and is more associated with geriatric conditions and diseases, especially atherosclerotic diseases. This supports the view that the chronic mucosal ischemia may cause colonic telangiectasia. While most of the patients are asymptomatic, a small number of patients show blood in the stool, mostly manifested as repeated small amount of bleeding in most patients and major bleeding in a small number of cases. Only a small number of patients are diagnosed using colonoscopy due to recurrent painless lower gastrointestinal bleeding or other reasons. Capillary dilatation is more common in the cecum and right colon, and major bleeding can occur.^[7,8]^ According to the diagnostic criteria of endoscopic diagnosis,^[11]^ the colonoscopy is characterized by spotted, patchy or spider-like bright red lesions or radially expanding capillary plexus, generally less than 10 mm in diameter with clear or blurred boundary, flat or slightly elevated, or manifested as diffuse dilatation of mucosal capillaries. Lesions can appear in 1 or several segments of the intestine, with or without active bleeding. The cause of bleeding may be due to the damage to the thin capillaries by an increase in intravascular pressure or by the intestinal contents. In this present case, there was no abnormality in the barium examination.

Colonoscopy is the main method of diagnosis of colonic telangiectasia, and the positive rate is greater than 90%. This procedure should be performed when there is no bleeding or a small amount of bleeding. Sometimes due to mild vascular lesions, it is difficult to identify colonic telangiectasia with endoscopic abrasion or suction injury. Hence, colonoscopy should be performed with careful observation. It has been reported that water-injection colonoscopy can improve the rate of diagnosis. It is a technique for colonoscopy by water injection, instead of traditional gas injection. When blood flows from the lesion into the filled lumen, it is easy to find the lesion under water-injection colonoscopy, which can facilitate further endoscopic treatment.^[12]^ For patients with active bleeding, angiography is feasible and can be used for diagnosis. When the bleeding rate is ≥0.5 mL/min, contrast agent spillover can be seen, and can help identify the lesion position accurately. If necessary, embolization can be performed simultaneously.^[13]^ When the diagnosis is difficult, and the continuous hemorrhage is endangering the patient's life, the laparotomy can be explored, and the colonoscopy can be used during the laparotomy to find the lesion.

Patients who are diagnosed with colonic telangiectasia but are asymptomatic do not need further treatment.^[14]^ It is usually recommended to monitor the color of stool and check the hemoglobin and fecal occult blood regularly.^[15]^ If patients have a small amount of bleeding, feasible endoscopic treatment (coagulation, laser, sclerotherapy, etc.) can be used.^[16]^ These methods are safe and convenient, cause less trauma and complications, and can be used repeatedly. They are especially suitable for patients with chronic diseases such as cardiopulmonary and elderly patients undergoing surgery.^[17]^ Estrogen-progesterone therapy has been used to achieve certain efficacy in treating colonic telangiectasia.^[18]^ The mechanism of this therapy may be to enhance the integrity of vascular endothelial cells and improve microcirculation and coagulation.^[18]^ However, surgical treatment is often required for patients with large bleeding volume and accurate lesion positioning.^[19]^ In conclusion, treatment of colonic telangiectasia should be personalized, depending on severity.

## Author contributions

J-AL, L-LZ and BL collected data; J-AL and D-QJ drafted the case report; Y-LZ supervised study, provided resources; All authors proof read and approved the report for publication.

**Data curation:** Jun-An Li, Dong-Qiang Jiang.

**Formal analysis:** Jun-An Li, Li-Li Zhong, Bo Li.

**Funding acquisition:** Yin-Long Zhao.

**Resources:** Yin-Long Zhao.

**Supervision:** Yin-Long Zhao.

**Writing – original draft:** Jun-An Li.

**Writing – review & editing:** Li-Li Zhong, Bo Li, Dong-Qiang Jiang, Yin-Long Zhao.
